# ASD-Net: a novel U-Net based asymmetric spatial-channel convolution network for precise kidney and kidney tumor image segmentation

**DOI:** 10.1007/s11517-024-03025-y

**Published:** 2024-02-08

**Authors:** Zhanlin Ji, Juncheng Mu, Jianuo Liu, Haiyang Zhang, Chenxu Dai, Xueji Zhang, Ivan Ganchev

**Affiliations:** 1 Department of Artificial Intelligence, North China University of Science and Technology, Tangshan, 063009 People’s Republic of China; 2 Department of Computing, Xi’an Jiaotong-Liverpool University, Suzhou, People’s Republic of China; 3 School of Biomedical Engineering, Shenzhen University Health Science Center, Shenzhen, Guangdong 518060 People’s Republic of China; 4 Telecommunications Research Centre (TRC), University of Limerick, Limerick, V94 T9PX Ireland; 5 Department of Computer Systems, University of Plovdiv “Paisii Hilendarski”, Plovdiv, 4000 Bulgaria; 6 Institute of Mathematics and Informatics, Bulgarian Academy of Sciences, Sofia, 1040 Bulgaria

**Keywords:** Kidney tumor, Image segmentation, Medical image analysis, Neural network, U-Net

## Abstract

Early intervention in tumors can greatly improve human survival rates. With the development of deep learning technology, automatic image segmentation has taken a prominent role in the field of medical image analysis. Manually segmenting kidneys on CT images is a tedious task, and due to the diversity of these images and varying technical skills of professionals, segmentation results can be inconsistent. To address this problem, a novel ASD-Net network is proposed in this paper for kidney and kidney tumor segmentation tasks. First, the proposed network employs newly designed Adaptive Spatial-channel Convolution Optimization (ASCO) blocks to capture anisotropic information in the images. Then, other newly designed blocks, i.e., Dense Dilated Enhancement Convolution (DDEC) blocks, are utilized to enhance feature propagation and reuse it across the network, thereby improving its segmentation accuracy. To allow the network to segment complex and small kidney tumors more effectively, the Atrous Spatial Pyramid Pooling (ASPP) module is incorporated in its middle layer. With its generalized pyramid feature, this module enables the network to better capture and understand context information at various scales within the images. In addition to this, the concurrent spatial and channel squeeze & excitation (scSE) attention mechanism is adopted to better comprehend and manage context information in the images. Additional encoding layers are also added to the base (U-Net) and connected to the original encoding layer through skip connections. The resultant enhanced U-Net structure allows for better extraction and merging of high-level and low-level features, further boosting the network’s ability to restore segmentation details. In addition, the combined Binary Cross Entropy (BCE)-Dice loss is utilized as the network's loss function. Experiments, conducted on the KiTS19 dataset, demonstrate that the proposed ASD-Net network outperforms the existing segmentation networks according to all evaluation metrics used, except for recall in the case of kidney tumor segmentation, where it takes the second place after Attention-UNet.

**Graphical Abstract:**

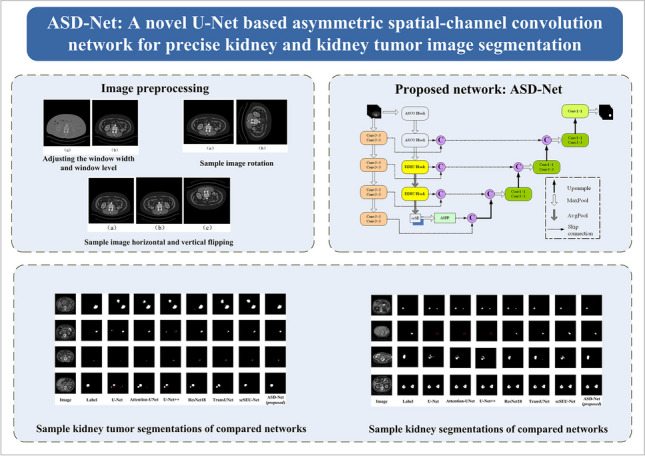

## Introduction

Recently, the cases of kidney tumors have increased worldwide [1, 2]. As one of the malignant tumors affecting the human urinary reproductive system, radical nephrectomy is the most commonly used method in clinical treatment. However, this procedure can result in post-operative issues, causing daily life inconveniences. Hence, kidney-preserving surgeries are commonly used in tumor treatments [3, 4], necessitating the accurate segmentation of the kidney and kidney tumor before surgery [5, 6]. However, challenges such as low contrast in the tissue details and the irregular shape of the kidney and tumor in abdominal scanning images can make precise segmentation difficult [7–10]. Artificial intelligence (AI) has shown potential in assisting clinical care as an effective way to detect and diagnose abnormal areas [11–13]. The most advanced AI algorithms can effectively distinguish between benign and malignant kidney masses on computed tomography (CT) scans [14–16]. However, the performance of AI algorithms still needs to be improved when distinguishing different subtypes of kidney cancer or evaluating tumors [17, 18].

With the significant progress made by deep learning (DL) in the fields of computer vision [19], natural language processing (NLP), and other areas, many problems in the field of medical image segmentation are being addressed using convolutional neural networks (CNNs) [20–22]. CNNs are essentially based on feature extraction algorithms that can learn the high-level semantic characteristics of various tissue/organ images from a large number of annotated CT images [23], realizing semantic segmentation of CT images [24, 25]. It is particularly noteworthy that the emergence of U-Net [26], a type of encoder-decoder structured neural network, has laid an important foundation for the development of subsequent biomedical image semantic segmentation networks. The introduction of U-Net marks that, in complex medical image analysis tasks, researchers are no more limited to traditional segmentation strategies, but can use the power of DL to extract and utilize richer and more complex feature information, thereby improving the accuracy and robustness of segmentation [27].

U-Net, proposed by Ronneberger et al. in [26], is a DL-based image segmentation network that is able to achieve precise image segmentation by utilizing an encoder-decoder architecture and skip connections. U-Net has shown excellent performance in many medical image segmentation tasks, including kidney tumor segmentation. Zhou et al. [28] proposed U-Net++ , which consists of a series of U-Nets with different depths and decoders. These decoders are densely connected at the same resolution through redesigned skip connections. Although with better segmentation performance, U-Net++ is a very complex network, requiring additional learnable parameters, and some of its components are redundant for specific tasks [29]. Oktay et al. [30] incorporated skip connections with focusing gates into a U-shaped structure for medical image segmentation, whereby an attention gate (AG) implicitly generates soft region suggestions, emphasizing relevant task features. Diakogiannis et al. [31] proposed a deep residual U-Net based network, named ResUNet-a, which uses a series of stacked residual units to replace the ordinary neural units as basic blocks, effectively deepening the network training layers. However, as the network depth increases, the training time also significantly increases. Researchers also consider introducing a self-attention mechanism in CNNs to improve network performance [29]. Çiçek et al. [32, 33] extended U-Net to three dimensions, proposing 3D U-Net. Chen et al. [34] proposed the DeepLab network, a DL network used for image segmentation, which utilizes dilated convolution and Atrous Spatial Pyramid Pooling (ASPP) structure to capture multi-scale contextual information, and can better handle complex image segmentation tasks such as kidney tumor segmentation. However, this method cannot allow the network to efficiently use the target features extracted. The abdominal CT images contain a large amount of complex background. If the features of the kidney and tumor images cannot be accurately extracted and efficiently used, irrelevant background information will greatly affect the final segmentation result.

This paper proposes a novel ASD-Net network, based on U-Net, which shows an enhanced segmentation performance. The main contributions of the paper are reflected in the following aspects:

1. An innovative combination of the asymmetric convolution and the concurrent spatial and channel squeeze & excitation (scSE) attention gate is proposed, forming a novel Adaptive Spatial-channel Convolution Optimization (ASCO) block for incorporation into U-Net. Without adding much to the computational complexity, this block enhances the network’s ability in complex pattern recognition and global context information understanding, thereby improving its image segmentation performance.
2. A novel Dense Dilated Enhancement Convolution (DDEC) block is proposed for incorporation into U-Net, in which the last 3 × 3 convolution of the dense convolution block is replaced with a dilated convolution, followed by a spatial and channel dual attention. This allows the network to effectively enlarge the receptive field, enabling it to capture a broader range of contextual information while keeping the number of parameters and computational complexity unchanged. In addition, it can also recalibrate the feature maps, enhance the important features, suppress the irrelevant features, and enhance the network’s segmentation performance.
3. To reduce the impact of noise, it is proposed to get rid of the skip connections at the top layer of the network in order to pay more attention to the learning of deep features. This way the lower-level features, which may contain a lot of noise and irrelevant information, are directly passed to the upper layers, thus positively affecting the segmentation performance of the network.
4. Inspired by N-Net [35], a dual-encoder/single-decoder backbone structure is utilized for the proposed ASD-Net network.

## Related work

### Image preprocessing

**Fig. 1:**
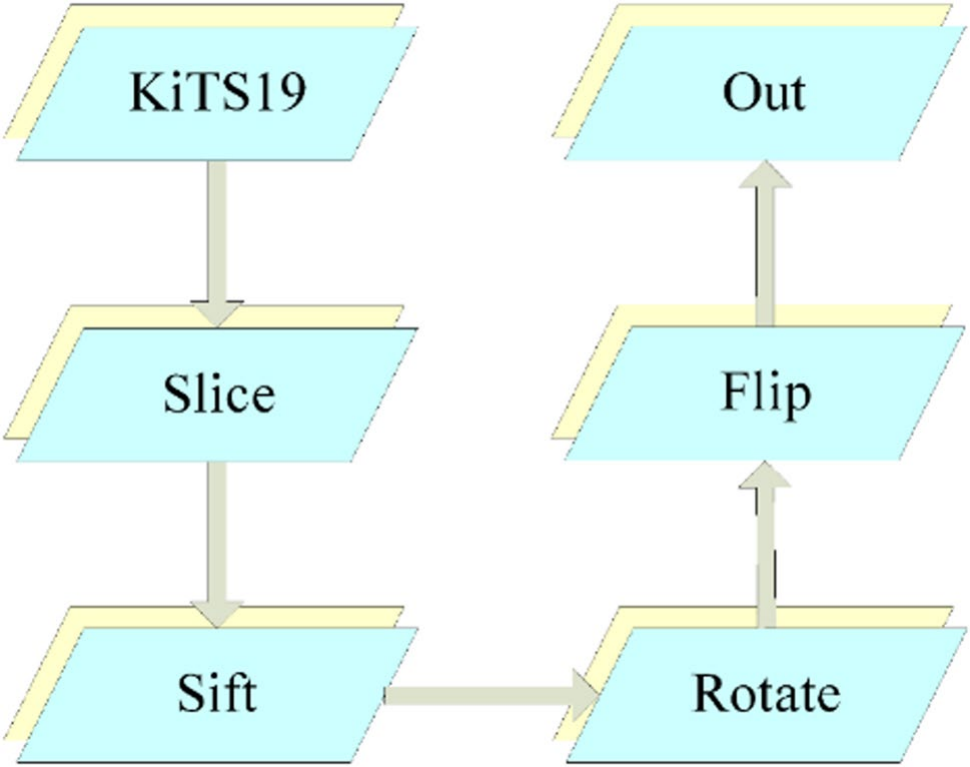
The utilized image preprocessing sequence

Data preprocessing and data augmentation are two key strategies for improving the training efficiency of DL networks. These strategies mainly improve the quality of the training sample set and make the networks more effectively adapted to the distribution of training data characteristics. Through data augmentation, one can generate more complex training data, which improves the network generalization ability and helps prevent overfitting [36]. Since the KiTS19 dataset, used in the conducted experiments, consists of three-dimensional (3D) images, but the elaborated segmentation network is two-dimensional (2D), the data were first converted from 3D to 2D. During the slicing conversion process, aimed at enhancing the details of the target tissue in the images, the window width and position were initially adjusted as follows: any CT value greater than 500 HU (Hounsfield units) was set to 500 HU, and any CT value less than –200 HU was set to –200 HU. After completing the slicing, pictures with kidneys or tumors were selected based on the marked images, invalid images were deleted, and random rotation, horizontal or vertical flipping on the training set’s images were performed before the network training, as shown in Fig. 1.

**Fig. 2:**
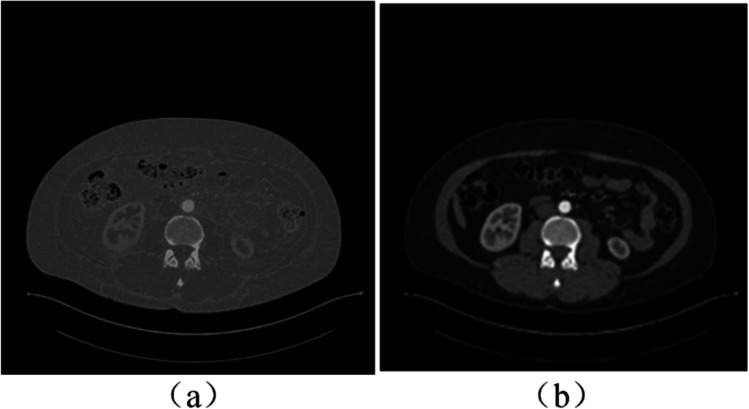
An illustrative result of adjusting the window width and window level: **a** before the adjustment; **b** after the adjustment

An illustrative example of adjusting the window width and window level is depicted in Fig. 2.

**Fig. 3:**
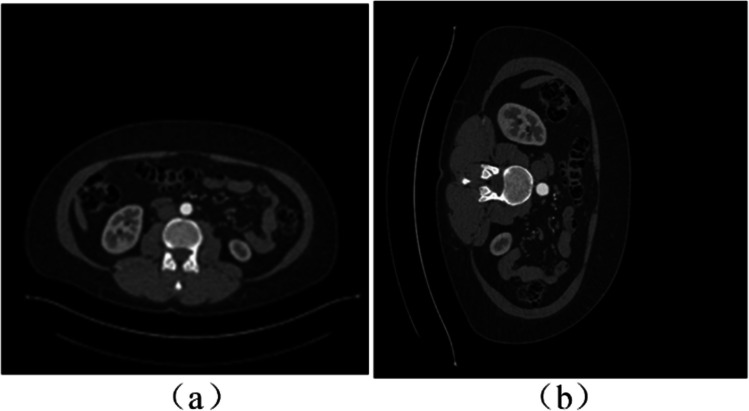
Sample image rotation: **a** original image; **b** clock-wise rotation of 90°

**Fig. 4:**
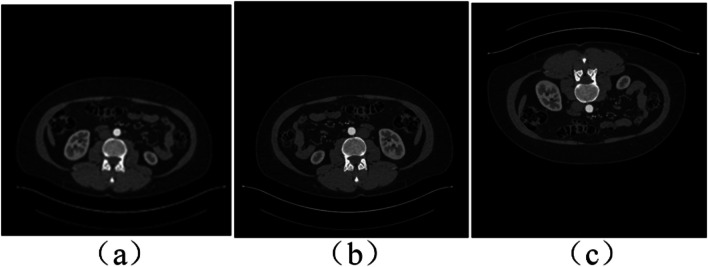
Sample image horizontal and vertical flipping: **a** original image; **b** horizontal flipping; **c** vertical flipping

A sample random rotation of an input image is shown in Fig. 3 and a sample random horizontal and vertical flipping (with a probability of 0.5) are illustrated in Fig. 4.

### U-Net

The classic architecture of U-Net is composed of two parts: (i) feature extraction, designed with inspiration from the VGG network [37, 38], where each pooling layer contains a specific scale, with a total of five different scales based on the original image; and (ii) upsampling, where each stage performs an upsampling operation and fuses information with the corresponding channels from the feature extraction part through skip connecting [39]. The encoder-decoder structure of U-Net allows it to capture and merge contextual information at different abstraction levels. This contextual information plays a key role in enhancing semantic understanding and accuracy of image segmentation.

### scSE attention mechanism

**Fig. 5:**
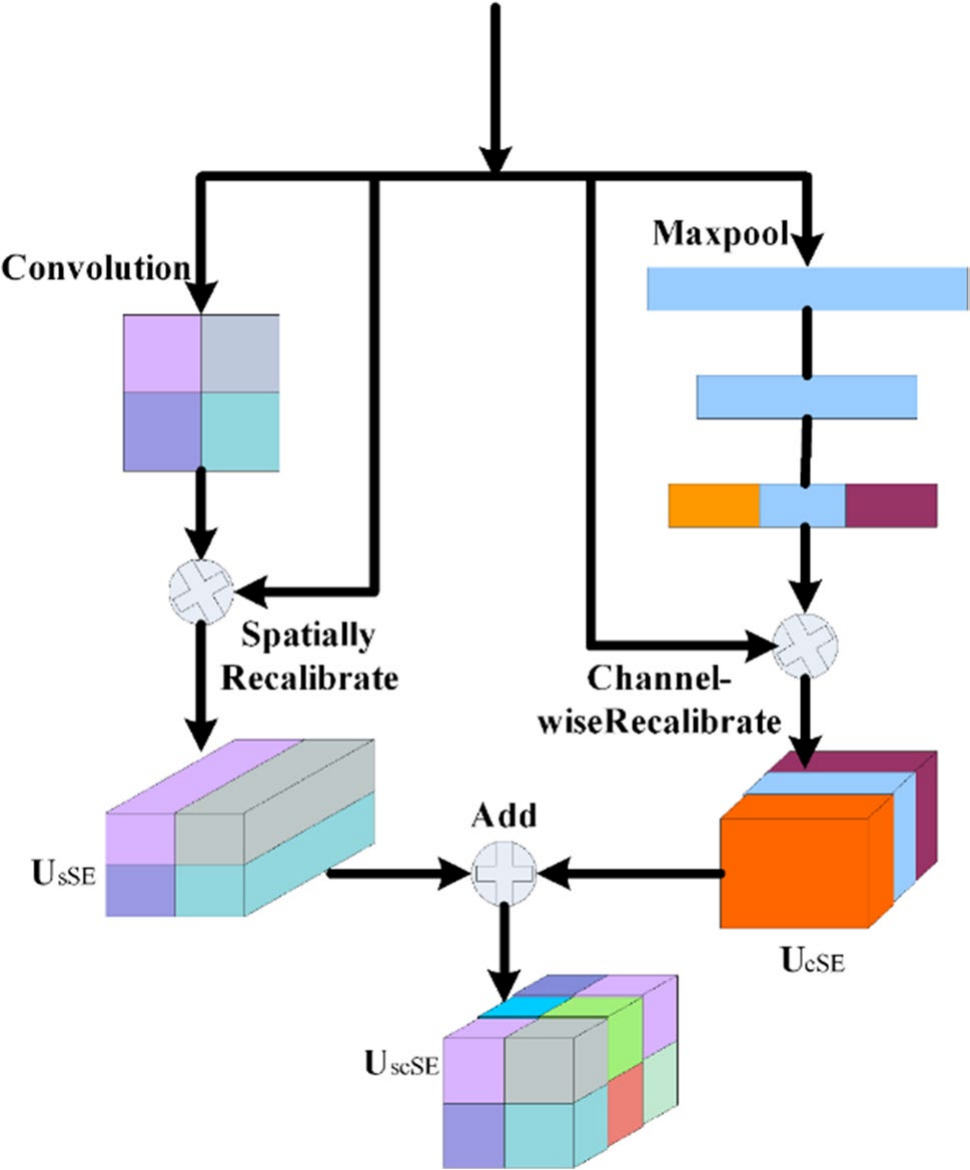
The scSE structure

The attention mechanism, initially used in machine translation, was quickly applied to the field of computer vision due to its outstanding performance. Today, the attention mechanism has become a common means for enhancing the neural networks [40]. By combining channel and spatial attention, the scSE attention mechanism [41], depicted in Fig. 5, significantly enhances the network learning ability. Its design strategy adjusts the importance of features at different levels, allowing a network to learn more valuable high-level features, while paying less attention to features that have less impact on the target task. This strategy allows to obtain richer spatial and channel information at the pixel level. The relative importance of attention in both dimensions is simultaneously adjusted, thus further improving the accuracy of downstream registration tasks [42].

### Loss functions

In the conducted experiments, described further in this paper, a combined Binary Cross Entropy (BCE)-Dice loss [43] is used as a loss function. This design of the loss function aims to take into account both the pixel-level classification loss and the region-level overlap loss in order to assess the segmentation performance more comprehensively.

Specifically, BCE is a commonly used loss function for binary classification problems, measuring the gap between the predicted output ${p}_{i}$ and the actual label ${y}_{i}$, as follows [44]:

1 $${L}_{BCE}=-\sum\nolimits_{i}({y}_{i}ln({p}_{i})+(1-{y}_{i})ln(1-{p}_{i}))$$

However, when dealing with imbalanced data—for instance, when most areas in the image are background with only a small part being tumors—it could cause a network to predict the majority categories, potentially “ignoring” the minority categories. Thus, when dealing with image segmentation tasks with uneven category distribution or large blank background areas, using BCE alone may result in poor predictive performance for minority categories.

To solve this problem, the Dice loss could be used in addition to the BCE loss. It is based on the Dice similarity coefficient (DSC), which is a commonly used metric to measure the similarity between two samples, especially suitable for dealing with category imbalance problems in medical image segmentation tasks. The Dice loss is defined in [45] as follows:

2 $${L}_{Dice}=1-2\frac{\sum_{i=1}^{N}{y}_{i}{p}_{i}}{\sum_{i=1}^{N}{y}_{i}^{2}+\sum_{i=1}^{N}{p}_{i}^{2}}$$

To attain precise segmentation of the kidney and tumors, and to overcome challenges such as slow network convergence, gradient disappearance during backpropagation, and class imbalance, a combined loss function made of these two loss functions is used for optimizing the network training, as follows:

3 $$L=1/2 {L}_{BCE}+{L}_{Dice}$$

The benefit of using this combined loss function is that when the prediction results are closer to the actual labels, or the overlap between the predicted area and the actual area is higher, the value of the BCE-Dice loss is smaller than that of each individual loss.

## Proposed network

### Overall structure

**Fig. 6:**
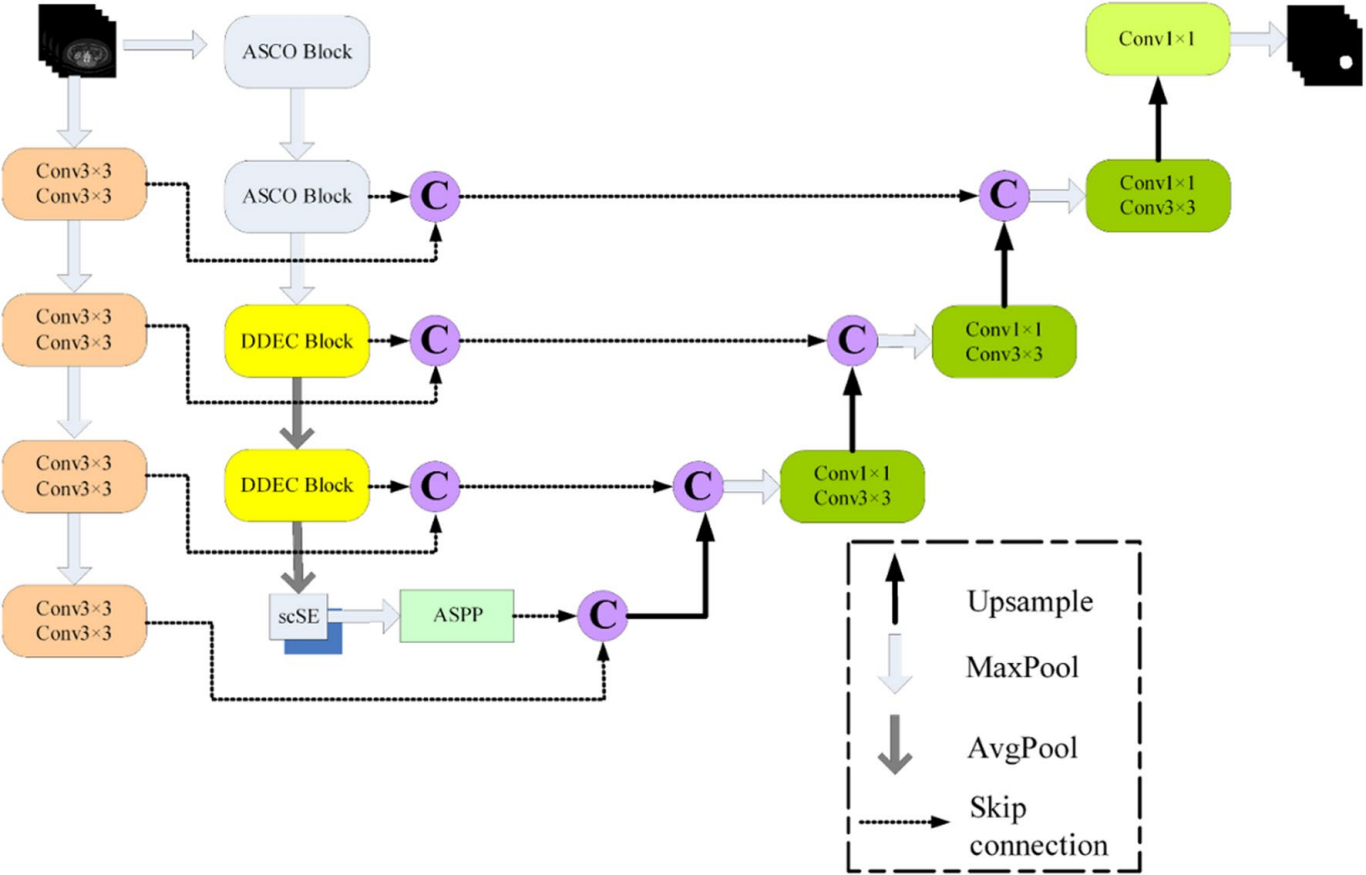
The proposed ASD-Net network

The ASD-Net network, proposed in this paper, is based on U-Net, with the following improvements, as shown in Fig. 6:

1. The incorporation of newly designed ASCO blocks into the U-Net structure allows to effectively capture the anisotropic properties of input images, thereby adapting the network to their inherent asymmetry and scale differences. This lays a solid foundation for subsequent feature extraction and fusion.
2. The addition of newly constructed DDEC blocks to U-Net not only enables drawing on the advantages of the dense connection structures in feature propagation and feature reusing but also effectively expanding the receptive field and deeply integrating and extracting different levels of features without increasing the computational complexity related to the use of dilated convolution, thereby improving the segmentation performance.
3. The incorporation of a scSE attention mechanism into U-Net endows it with an ability to analyze and process contextual information in input images more deeply. The scSE mechanism significantly enhances the network’s ability to capture key spatial and channel information, thus further improving the segmentation results.
4. The addition of an extra encoding layer in the U-Net left side and the removal of the top skip connection between the encoding and decoding layers allows emphasizing on the network’s extraction and utilization of advanced features by fusing features at different levels, thus further enhancing the network’s segmentation performance and its ability to recover details.
5. The incorporation of an ASPP pooling pyramid module [34] between the encoding and decoding layers, for performing multi-scale spatial sampling on the input in parallel, enables extracting rich global contextual information that positively affecting the network’s segmentation ability at multiple scales.

These U-Net improvements are described in the following subsections in greater detail.

### ASCO block

**Fig. 7:**
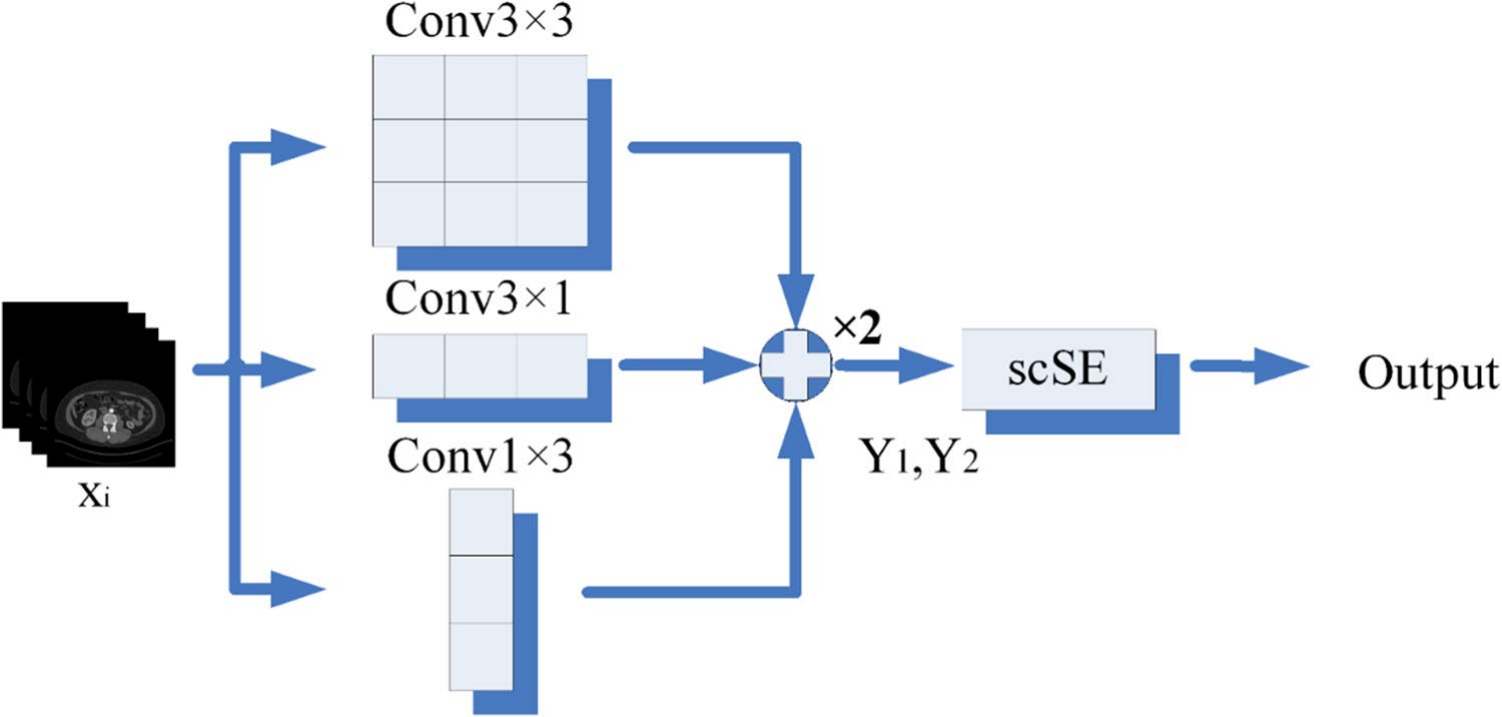
The proposed ASCO block

The design of the ASCO block aims to more effectively extract and use feature spatial and channel information in order to improve the segmentation performance. The ASCO core idea is to use scSE with asymmetric convolution (Fig. 7).

The scSE component acts on the spatial and channel axes of the input feature maps, generating two activation maps, and then multiplies these with the input feature map to recalibrate the features. This adaptive adjustment allows the proposed ASD-Net network to obtain richer spatial and channel information at the pixel level.

In addition, the ASCO block uses asymmetric convolution (vertical and horizontal) to better integrate spatial and channel information and deeply mine the correlation between features. For this, ASCO integrates complex asymmetric convolution operations and attention gates into a standard convolution operation. This design helps reduce computational complexity in practical applications and improve network runtime efficiency.

The design of the ASCO block is well-grounded theoretically. Existing research has shown that for parallel processing of space and channels, both spatial and channel information play significant roles in image processing tasks [46]. Moreover, asymmetric convolution can better extract and integrate spatial and channel information, which is also supported by related research [47].

The effectiveness of the ASCO block is validated through ablation study experiments, as described further in Subsection 4.4.4.

For image ${x}_{i}$, the calculation formulae used by ASCO at the first and second layers of asymmetric convolution are the following:

4 $${Y}_{1}=BN({x}_{i}*{W}_{3\times 3}+{b}_{3\times 3}+{x}_{i}*{W}_{3\times 1}+{b}_{3\times 1}+{x}_{i}*{W}_{1\times 3}+{b}_{1\times 3})$$

5 $${Y}_{2}=BN({Y}_{1}*{W}_{3\times 3}+{b}_{3\times 3}+{Y}_{1}*{W}_{3\times 1}+{b}_{3\times 1+}{Y}_{1}*{W}_{1\times 3}+{b}_{1\times 3}$$

where BN denotes batch normalization operation, ${W}_{i\times j}$ denotes convolution operation of i × j, and ${b}_{i\times j}$ denotes the bias of i × j.

Then, following the scSE operation, the final output *Y* is obtained as follows:

6 $${Y}_{cSE}={Y}_{2}\times Sigmod[{W}_{1}*({W}_{2}*Avg({Y}_{2}))]$$

7 $${Y}_{sSE}={Y}_{2}\times Sigmod({Conv}_{1\times 1}({Y}_{2}))$$

8 $$Y={Y}_{cSE}+{Y}_{\text{sSE}}$$

where ${Y}_{cSE}$ represents the output obtained through the Spatial Squeeze and Channel Excitation (cSE) part of the concurrent spatial and channel squeeze & excitation (scSE) mechanism. ${Y}_{sSE}$ represents the output obtained through the Channel Squeeze and Channel Excitation (sSE) part of the concurrent scSE mechanism, and ${W}_{1}$ and ${W}_{2}$ being the weights of two fully connected layers.

### DDEC block

**Fig. 8:**
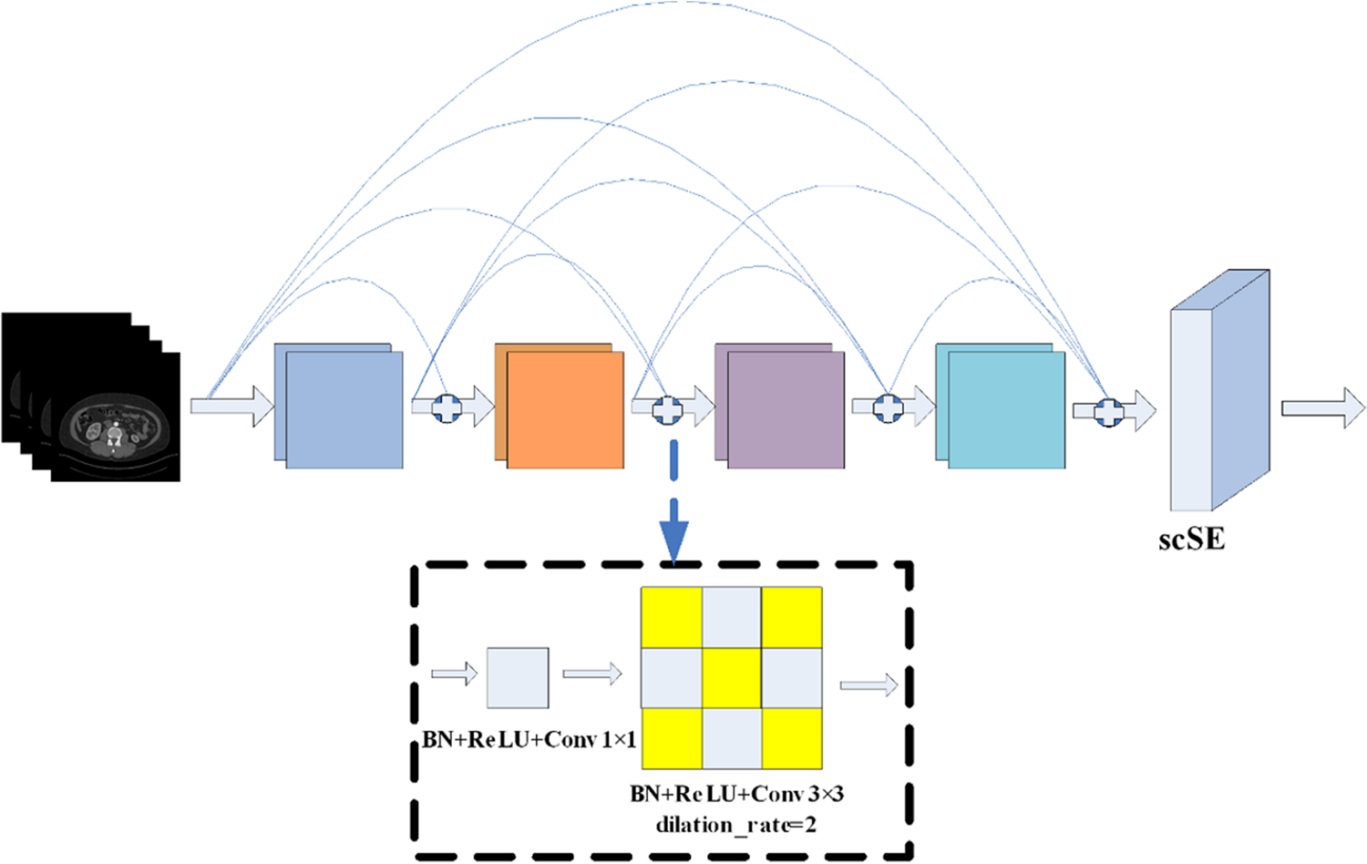
The proposed DDEC block

The proposed DDEC block enhances traditional dense connections by replacing the original 3 × 3 standard convolution with dilated convolution and incorporating an scSE attention gate (Fig. 8). This seemingly simple replacement led to an unexpected result, having a significant impact on the network segmentation performance.

Firstly, the DDEC block can effectively expand the receptive field of the network by using dilated convolution, without a need of additional parameters or extra computational complexity. This is crucial for capturing and understanding substantial contextual information in images, particularly for the target task of kidney and kidney tumor segmentation. A large receptive field can help the network acquire a broader context, thus achieving a better balance between details and global information. At the same time, the addition of the scSE attention gate allows the network to perform “squeeze-and-excitation” operations in both the spatial and channel dimensions. This allows the network to emphasize not only on the important channels but also to highlight key areas in the input feature map.

Secondly, compared to traditional dense connections, the proposed DDEC block retains an advantage of enhancing feature propagation and reuse. This allows each convolution layer to receive feature maps from all previous layers, conducive to acquiring richer feature information and ensuring better gradient backpropagation, thus improving the network training stability.

Finally, the incorporation of the DDEC block makes the network more effective in acquiring multi-scale features. Dilated convolution can obtain features of multiple scales while keeping the number of parameters unchanged, thus allowing the network to handle kidneys and kidney tumors with large-scale changes more effectively.

### Dual-encoder/single-decoder backbone

The inspiration about this improvement comes from the N-Net network [38], where two parallel paths of the dual encoder are interconnected layer-by-layer, through standard skip connections. As some information might be lost during the encoding and decoding, N-Net introduces a dual encoder network in order to reduce such losses. However, while applying this strategy, we introduced another innovation related to the removal of the top skip connection between the original encoding and decoding layers. Such dual encoder network not only deepens the network depth but also integrates more comprehensive information [38].

In the traditional U-Net architecture, features at all levels are treated equivalently and passed and fused through skip connections. However, we observed that the top-level feature information mainly contains global, low-level semantic information, which contributes little to the details of the segmentation task. Therefore, we chose to remove the top skip connection between the encoding and decoding layers, thereby putting more attention and resources on higher-level, more distinctive, and detailed features.

In the proposed network, the first encoder utilizes maximum average pooling, two 3 × 3 convolution layers, and the Rectified Linear Unit (ReLU) activation function to extract features. In contrast, the second encoder, composed of an ASCO block, a DDEC block, and an scSE attention mechanism [45], is responsible for extracting and reconstructing more complex features. These two encoders are interconnected through skip connections, facilitating the full utilization and integration of features at different levels and scales. This fusion method helps improve the network’s expressiveness and prediction accuracy and also makes the network more robust when dealing with complex tasks.

This meticulous design enables the proposed ASD-Net network to capture more fine-grained details and more intense semantic information, thus achieving a significant performance improvement in image segmentation tasks.

## Experiments and results

### Datasets

To evaluate segmentation performance of the proposed ASD-Net network in comparison to other existing networks, experiments were conducted on a dataset, sourced from the 2019 Kidney and Kidney Tumor Segmentation Challenge (KiTS19), which is a diverse dataset in terms of the voxel dimensions, contrast timing, table signature, and scanner field of view [48]. As stated in its original paper [49], this dataset was reviewed and approved by the Institutional Review Board at the University of Minnesota as Study 1611M00821. Additionally, the KiTS19 dataset is made available under the CC BY-NC-SA (Creative Commons Attribution-NonCommercial-ShareAlike) license, as of its publication date. We diligently adhered to the terms of this license throughout our research process to ensure compliance.

The KiTS19 dataset includes both original abdominal CT images and label images manually annotated by doctors. The dataset incorporates a range of different cases from 210 patients, thereby increasing its complexity and diversity, which in turn provides a more challenging environment for network training. In the experiments, the images of the first 170 patients were selected to form the training set, the images of the next 20 patients formed the validation set, and the images of the last 20 patients comprised the test set. All utilized CT scans contained kidney or tumor images, with a resolution of 512 × 512 pixels.

Due to variations in the number of 2D images extracted per patient, we selectively chose for analysis the images containing either kidneys or tumors. Consequently, in the tumor segmentation experiments, we utilized 4857 images for network training, 305 images for validation, and 294 images for testing. In the case of kidney segmentation experiments, we employed 13840 images for network training, 766 images for validation, and 842 images for testing. This selection ensured utilization of only those images, which are relevant to the specific tasks under consideration, while accounting for the uneven distribution of data contributed by each patient. Such a strategy helped maintain consistency in network training and evaluation, enhancing the interpretability and comparability of experimental results.

Although the KiTS19 dataset was the only one used in the experiments, its richness and diversity of patient cases ensured that the elaborated network has a good generalization ability. Firstly, this dataset covers a wide variety of cases with different disease characteristics and stages, thus training the network to handle various situations. Secondly, a strict training/validation/testing division strategy was adopted to avoid overfitting and conduct evaluations on unseen test sets, ensuring an accurate estimate of the network’s generalization ability. Finally, the superior segmentation performance demonstrated by the proposed ASD-Net network further substantiates its strong generalization ability when handling unseen, real medical image data.

### Evaluation metrics

In the experiments, four evaluation metrics, including Intersection over Union (IoU), DSC, recall, and precision, were used to quantitatively evaluate the performance of the proposed ASD-Net network, compared to other networks.

IoU is a widely used evaluation metric for image segmentation that measures the degree of overlap between the detected segmentation mask and the ground truth mask, calculated as follows:

9 $$IoU=\frac{TP}{TP+FP+FN}$$

where TP (true positives) represents the number of correctly identified pixels as being part of an object (i.e., a kidney/tumor in our case), FN (false negatives) represents the number of incorrectly identified pixels as being not part of an object, and FP (false positives) represents the number of incorrectly identified pixels as being part of an object.

DSC is another widely used evaluation metric for image segmentation, which describes the degree of similarity between the detected segmentation and its corresponding ground truth, calculated as follows:

10 $$DSC=\frac{2TP}{2TP+FP+FN}$$

Recall refers to the proportion of the boundary pixels in the ground truth that are successfully detected by a network, calculated as follows:

11 $$Recall=\frac{TP}{TP+FN}$$

Precision refers to the proportion of the boundary pixels in the segmentation corresponding to the boundary pixels in the ground truth, calculated as follows:

12 $$Precision=\frac{TP}{TP+FP}$$

### Experimental setup

The hardware configuration used in the experiments included an InterCore i5-12490 processor with a main frequency of 3.0 GHz and a single NVIDIA RTX3060 graphics card with 12 GB of memory. To ensure normal training, the following settings were used: a batch size set to 4, the number of epochs set to 100, validation was performed on every epoch, the adaptive moment estimation (Adam) optimizer was used to train the network, the initial learning rate was set to 1 × 10^−4^, the decay coefficient was set to 1 × 10^−4^ to prevent overfitting, momentum was set to 0.9, the minimum learning rate was 1 × 10^−5^, and the network structure was implemented by Pytorch.

### Results and analysis

#### Kidney segmentation comparison with classic networks

First, the kidney segmentation performance of the proposed network was compared to that of classic segmentation networks, such as U-Net, Attention-UNet, U-Net++ , ResNet18, TransUNet, and scSEU-Net. The obtained results are presented in Table 1 (the best result on each metric is shown in bold). Based on these, it is clear that the proposed ASD-Net network outperformed all other networks according to all evaluation metrics. More specifically, the second-best performing network (scSEU-Net) was surpassed by 1.44 percentage points on IoU, 0.84 percentage points on DSC, and 0.75 percentage points on recall, and by 0.14 percentage points on precision, where U-Net took the second place.

**Table 1:** Kidney segmentation performance comparison of ASD-Net with classic networks

Networks	IoU (%)	DSC (%)	Recall (%)	Precision (%)
U-Net	90.08	94.58	92.74	96.90
Attention-UNet	91.45	95.35	94.24	96.85
U-Net++	90.75	94.96	93.87	96.46
ResNet18	91.02	95.03	94.25	96.35
TransUNet	91.04	95.11	94.32	96.32
scSEU-Net	91.91	95.60	95.32	96.20
ASD-Net (*proposed*)	**93.35**	**96.44**	**96.07**	**97.04**

The best results are shown in bold

**Fig. 9:**
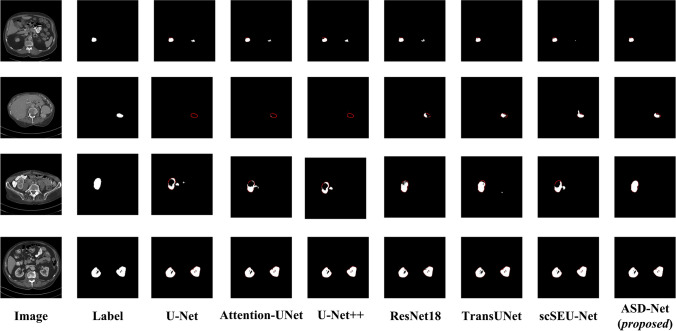
Sample kidney segmentations performed by the compared networks (the red contour lines highlight the regions that should be successfully segmented)

While the quantitative comparison highlights the performance improvement achieved by the proposed network, it may not fully convey its advantages. Thus, in Fig. 9, a visual comparison is provided of segmentation results achieved by different networks in segmenting kidneys on the KiTS19 dataset.

#### Kidney tumor segmentation comparison with classic networks

Then, the proposed network was compared in terms of the kidney tumor segmentation with the classic segmentation networks, participating in the previous experiment. The obtained results are shown in Table 2 (the best result on each metric is shown in bold). Again, the proposed ASD-Net network outperformed all other networks according to all evaluation metrics, except for recall, where it took the second place after Attention-UNet. More specifically, the second-best performing network (scSEU-Net) was surpassed by 4.98 percentage points on IoU, 4.20 percentage points on DSC, and 3.65 percentage points on precision, which demonstrates the higher superiority of ASD-Net in terms of the kidney tumor segmentation. While Attention-UNet outperforms the proposed network in terms of recall, ASD-Net excels  it by far on commonly used segmentation metrics such as IoU, DSC, and precision, highlighting its superiority in specific tasks. Higher values of IoU and DSC achieved by ASD-Net indicate that it is more accurate at the pixel level, while high precision demonstrates its ability in predicting positive instances. This balanced performance makes ASD-Net more competitive in overall segmentation tasks. Attention-UNet may be more suitable for scenarios emphasizing high recall, such as initial screening or rapid detection in medical imaging, where a preliminary rough segmentation can be efficiently accomplished by a high-recall network. However, in the final detailed segmentation, the proposed ASD-Net network remains more competitive. Therefore, it is capable of solving (to some extent) the problem of separating small tumor areas from kidneys and can compensate for the problems of missed detection due to incorrect segmentation of the lesion location performed by other networks, thus playing an auxiliary guiding role in the clinical diagnosis of kidney tumors.

**Table 2:** Kidney tumor segmentation performance comparison of ASD-Net with classic networks

Networks	IoU (%)	DSC (%)	Recall (%)	Precision (%)
U-Net	64.49	76.93	81.80	76.59
Attention-UNet	69.20	80.29	**91.85**	74.21
U-Net++	65.94	77.95	85.26	74.83
ResNet18	67.45	79.36	84.15	78.10
TransUNet	64.86	77.01	82.44	76.53
scSEU-Net	70.48	81.02	85.57	80.54
ASD-Net (*proposed*)	**75.46**	**85.22**	88.55	**84.19**

The best results are shown in bold

**Fig. 10:**
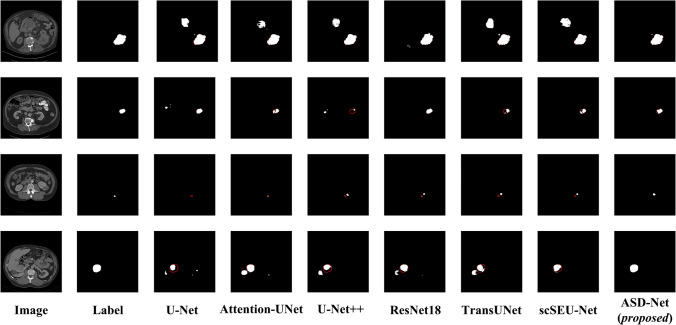
Sample kidney tumor segmentations performed by the compared networks (the red contour lines highlight the regions that should be successfully segmented)

Figure 10 shows a visual comparison of results achieved by different networks in segmenting kidney tumors on the KiTS19 dataset (the white area represents the tumor segmentation result). As can be seen from Fig. 10, traditional classic networks such as U-Net and U-Net ++ have rough segmentation of the edges of complex-shaped, small-volume tumor targets, and there are cases of erroneous segmentation. In contrast, the proposed ASD-Net network performs well in segmenting small and irregular lesion areas.

#### Kidney and kidney tumor segmentation comparison with state-of-the-art networks

Next, the proposed network was compared in terms of both kidney segmentation and kidney tumor segmentation with state-of-the-art networks, based on their DSC results reported in the corresponding literature sources, as shown in Table 3 (the best DSC result achieved in each task is shown in bold). The proposed ASD-Net network outperformes all state-of-the-art networks by scoring 2.82 and 0.05 percentage points more than the second-best performing network in kidney segmentation and kidney tumor segmentation, respectively, according to DSC.

**Table 3:** Kidney and kidney tumor segmentation performance comparison of ASD-Net with state-of-the-art networks

Networks	DSC (%) for kidney tumor segmentation	DSC (%) for kidney segmentation
RAU-Net [50]	76.75	96.19
LC-Unet [51]	78.91	96.39
MSVRL+nnU-Net [52]	74.50	95.98
O2M-UDA [53]	75.40	92.30
SLM-SA [54]	82.40	-
MedNeXt [55]	-	91.02
ASD-Net (*proposed*)	**85.22**	**96.44**

The best results are shown in bold

#### Ablation study

To verify the effectiveness of each module added to the baseline (U-Net) when designing the proposed ASD-Net network, ablation study experiments were conducted separately for kidney segmentation and kidney tumor segmentation. In these experiments, we incrementally added first the ASCO block and then the DDEC block, then removed the original top-level skip connection, added an ASPP module, and finally added a dual encoder to form the final network, all under the same experimental environment. The results obtained in the kidney segmentation are shown in Table 4, whereas Table 5 presents the obtained results in the kidney tumor segmentation (the best result on each metric is shown in bold). As can be seen from these tables, in the first step, the addition of ASCO block to U-Net led to improving the values of all evaluation metrics, except for precision. The additional incorporation of the DDEC block in the second step further improved the values of all evaluation metrics, except for recall in case of kidney tumor segmentation. The removal of the original top-level skip connection was beneficial for all metrics, except for precision in kidney segmentation and recall in kidney tumor segmentation. The addition of the ASPP module improved all evaluation metrics, except for recall in kidney tumor segmentation. The last step of adding the dual encoder, by which the final network was formed, allowed to improve all metrics, except for precision (according to this metric, the result of the previous step was the best one among all others). By using majority voting (3 out of 4 evaluation metrics reported the best results), it was decided to promote the result of the last step as a novel ASD-Net network, which is the subject of this paper.

**Table 4:** Ablation study results for kidney segmentation

Networks	IoU (%)	DSC (%)	Recall (%)	Precision (%)
U-Net	90.08	94.58	92.74	96.90
+ ASCO	91.18	95.20	94.28	96.50
+ DDEC	91.71	95.50	94.60	96.80
-cat	91.87	95.57	94.95	96.58
+ ASPP	92.61	96.00	95.20	**97.11**
ASD-Net (*proposed*)	**93.55**	**96.44**	**96.07**	97.04

The best results are shown in bold

**Table 5:** Ablation study results for kidney tumor segmentation

Networks	IoU (%)	DSC (%)	Recall (%)	Precision (%)
U-Net	64.49	76.93	81.80	76.59
+ ASCO	69.29	80.35	88.19	76.52
+ DDEC	72.70	82.26	88.15	79.54
-cat	73.99	83.49	87.66	82.12
+ ASPP	74.80	84.37	86.11	**85.26**
ASD-Net (*proposed*)	**75.46**	**85.22**	**88.55**	84.19

The best results are shown in bold

## Conclusions and future directions

To address the issue of inaccurate kidney and kidney tumor segmentation, this paper has proposed improvements to the U-Net network structure, leading to a more advanced network, named ASD-Net. Firstly, an encoder of context-aware features was designed, by combining efficient attention channels and the newly designed ASCO and DDEC blocks, and depth and spatial information, to extract multi-scale feature images. Then, based on the U-Net structure, additional encoding layers were added and connected by skip connections in order to optimize feature extraction and fusion, thus further enhancing detail restoration capabilities of the proposed network. Subsequently, a combined BCE-Dice loss was utilized to mitigate the issue of unbalanced positive and negative samples and to enhance the accuracy of boundary segmentation. The experimental results demonstrated that even the hard-to-segment areas of kidneys and kidney tumors can be completely delineated, exhibiting smooth boundary contours.

While ASD-Net has made significant strides in addressing kidney and kidney tumor segmentation, it is worth also noting its limitations, which provide valuable directions for future improvements.

Firstly, ASD-Net may face challenges in handling small regions, particularly when these are covered by overlapping structures, leading to less accurate segmentation results. Additionally, despite the network’s outstanding performance in segmentation, its processing speed may not be ideal, limiting its applicability in real-time applications or large-scale dataset processing. In addition to the mentioned limitations, another drawback of ASD-Net is its inability to directly process 3D graphics. The current workflow requires the conversion of 3D to 2D graphics before inputting them into the network for segmentation, potentially causing information loss and introducing complexity and inaccuracies when dealing with 3D medical images.

Future research directions will focus on addressing these limitations while further enhancing the performance of ASD-Net. Firstly, efforts will be directed towards optimizing segmentation time to meet the demands of real-time applications and large-scale dataset processing. Secondly, there will be a concentrated effort to improve ASD-Net’s ability to handle segmentation of small areas, potentially through the introduction of more intelligent context information capture or specifically designed structures. Simultaneously, future plans include also enhancing the robustness of the network, reducing dependency on parameter selection, and increasing its universality. Future developments may also involve expanding ASD-Net’s support for multi-modal images and exploring the integration of other advanced technologies, such as transfer learning, reinforcement learning, or self-supervised learning, to further improve the network’s performance and applicability.
